# Invariant Natural Killer T (iNKT) Cells in HAART-Treated, HIV-Positive Patients with Bone and Cardiovascular Impairment

**DOI:** 10.1371/journal.pone.0110287

**Published:** 2014-10-17

**Authors:** Camilla Tincati, Matteo Basilissi, Elisabetta Sinigaglia, Esther Merlini, Giovanni Carpani, Antonella d’Arminio Monforte, Giulia Marchetti

**Affiliations:** 1 Department of Health Sciences, Clinic of Infectious Diseases and Tropical Medicine, “San Paolo” Hospital, University of Milan, Milan, Italy; 2 Blood Transfusion Unit, “San Paolo” Hospital, Milan, Italy; New York University, United States of America

## Abstract

**Background:**

Invariant Natural Killer T (iNKT) cells represent a determinant in the course of infections and diseases, however, their role in the pathogenesis of non-infectious co-morbidities in HIV-positive patients is unknown.

**Methods:**

Flow cytometry was used to investigate iNKT cell frequency, phenotype and function in HIV-infected patients on HAART with bone and/or cardiovascular disorders and in HIV-positive controls free from co-morbidities.

**Results:**

iNKT cells from subjects with bone and cardiovascular impairment expressed high levels of CD161 and predominantly secreted TNF. iNKT cells from individuals with bone disease alone did not show any distinctive phenotypical or functional characteristics. The functional capacity of iNKT cells in patients with cardiovascular disorder was impaired with no cytokine release upon stimulation.

**Conclusion:**

iNKT cells may have a role in non-infectious co-morbidities in treated HIV disease, possibly through the exacerbation of inflammation. Further studies are needed to investigate iNKT cells in the pathogenesis of non-communicable disorders in HIV infection.

## Introduction

HIV-positive patients on virologically-suppressive treatment are at risk of non-infectious co-morbidities [1]. Indeed, HIV infection is characterized by a state of persistent inflammation/immune activation [2]–[4] known to predict clinical progression [5]–[8]; such abnormalities are a distinctive characteristic of a senescent immune system [9] which may accelerate the aging process in the HIV-infected population [10]–[12]. In keeping with these observations, HIV-positive patients with reduced Bone Mineral Density (BMD) have been shown to feature a hyperactivated peripheral T-cell phenotype [13]; similarly, HIV-infected subjects with increased carotid Intima Media Thickness (IMT) and/or positive history for cardiovascular disease show expansion of activated CD8+CD38+ cells [14]–[17]. Most interestingly, T-cell activation has been described an independent risk factor for osteopenia/osteoporosis [13] and subclinical carotid abnormalities [17], [18].

While T-cell and monocyte activation has been extensively investigated in the setting of non-infectious co-morbidities in course of treated HIV disease and postulated as a possible marker of “immunosenescence” in this patient population, very few studies have investigated the role of other lymphoid cells in the pathogenesis of non-infectious co-morbidities in HIV-infected subjects.

Invariant natural killer T (iNKT) cells are a rare population of T cells that possess qualities of both the innate and adaptive arms of the immune response; iNKT cells recognize glycolipid antigens presented by the non-classical MHC molecule CD1d [19]–[21] and represent key components in the pathogenesis of many clinical conditions [22]–[30].

Frequency and function of iNKT cells are impaired in the course of HIV disease [31]–[35]. Indeed, iNKT cells express both CD4 and the CXCR4/CCR5 co-receptors, thus representing a target for the virus [36]. Accordingly, the CD4+ iNKT cell subset is preferentially depleted in HIV disease [31], [33], [34], [37] with a parallel loss of IL-4 and IFN-γ production [38] and such defects are only partially restored by HAART [38]–[40]. Interestingly, the production of Th1 cytokines from iNKT cells, such as IFN-γ and TNF has been inversely correlated with cell surface expression of CD161 [32], thus suggesting that this molecule may represent a marker of iNKT exhaustion in course of HIV [32].

iNKT cells have also been implicated in atherogenesis [41]–[43]; indeed, in the mouse model, CD4+ iNKT cells are recruited to the atherosclerotic lesions within arterial walls [44], [45] and contribute to the formation of fatty streaks [45]–[47]. Of note, CD1d is also expressed in human atherosclerotic lesions [48], [49] and lower frequencies of iNKT were found in circulating blood of patients with symptomatic atherosclerosis [49], [50]. More specifically, iNKT cells infiltrating human atherosclerotic tissue express CD4, CD161 and produce IFN-γ [51] and seem to be implicated in plaque stability through the interaction with vascular smooth muscle cells [51].

Furthermore, in the mouse model, α-GalCer-activated iNKT cells have been demonstrated to increase the frequency of osteoclast progenitor cells and favour their maturation into osteclasts [52]. The pro-osteocalstogenic effect of iNKT cells is positively regulated by TNF, while IFN-γ negatively affects this process [52]. While specific iNKT subsets have been linked to overt cardiovascular disease in humans [49], [50], to our knowledge, clinical findings on iNKT and osteopenia/osteoporosis in humans have yet to be described.

In this report we investigated iNKT cell frequency, phenotype and function in HIV-positive patients on virologically-suppressive HAART with bone and/or cardiovascular impairment.

Our study is the first to show that iNKT cells from HIV-infected individuals with cardiovascular and bone co-morbidities express high levels of CD161 and predominantly secrete TNF, suggesting a role in the pathogenesis of immunosenescent disorders in treated HIV infection.

## Materials and Methods

### Study Patients

We consecutively recruited HIV-positive patients on virologically-suppressive HAART (HIV-RNA<40 cp/ml) with available Bone Mineral Density (BMD; Dual-energy X-ray Absorptiometry-DXA) and carotid Intima Media Thickness (IMT; ultrasonography) measures for the screening of non-communicable disorders. The research has been approved by the Ethical Committee of San Paolo Hospital, Milan. All study participants provided written informed consent. All study participants provided written informed consent.

Bone disease was defined by the presence of osteopenia or osteoporosis upon DXA scan: According to WHO criteria, osteopenia and osteoporosis were defined by T-scores at the lumbar spine and/or femoral neck that were <−1 SD and ≥−2.5 and <−2.5, respectively [53]. Cardiovascular disease was defined by either right and/or left carotid IMT>1 mm [54], [55] or presence of a carotid plaque upon ultrasonographic evaluation.

On the basis of lumbar/femoral BMD and carotid IMT values, patients were divided into the following 4 groups: a) Double Positive (DP) patients with both bone and cardiovascular impairment (n = 10); subjects with Bone Disease (BD) (n = 10) or Cardiovascular Disease (CD) (n = 10) alone; Double Negative (DN) patients with neither bone nor cardiovascular disorders (n = 10).

### Human Lymphocyte separation and stimulation assays

Human peripheral blood (8 mL) was collected into EDTA tubes and PBMCs were isolated using ficoll-paque (Biocoll separating solution, BIOSPA). Cells of all patients were cultured in R10 medium alone (composition per 100 mL R10∶88 mL RPMI, 10 mL fetal bovine serum, 1 mL [100 UI/mL] L-glutamine, and 1 mL [100 UI/mL] penicillin/streptomycin; Euroclone, Italy) (unstimulated, US), or in medium supplemented with Phorbol 12-myristate 13-acetate (PMA, 50 ng/mL; Sigma-Aldrich, Milan, Italy)/ionomycin (500 ng/mL; Sigma-Aldrich, Milan, Italy). In a subgroup of 20 patients PBMCs were stimulated also with α-GalactosylCeramide (α-GalCer, 200 ng/mL; Enzo Life Sciences, NY, USA). Dose response curves were performed to determine the optimal concentration of both stimuli.

### Flow Cytometry

#### Parameters for inclusion of Natural Killer T surface phenotype and functional data

As iNKT cell levels are very low in peripheral blood, particularly during HIV infection, 1 million of total events (and never less than 500,000) were acquired for each sample. Moreover, as previously performed [32] a minimum of 20 events collected within the iNKT gate was required for the data to be considered for functional data analysis.

#### Surface staining

For the measurement of iNKT cell frequency and CD161 expression, freshly-isolated PBMCs were incubated with CD1d-tetramer-α-GalCer-PE (α-GalCer) (Proimmune, Oxford, UK), anti-CD3-PE Cy7 (Beckman Coulter, Fullerton, California, USA), anti-CD161-FITC (Mylteni Biotec, Bologna, Italy) anti-Vα24-biotin (Beckman Coulter, Fullerton, California, USA), for 30 minutes at 4°C. Cells were then washed with buffer (PBS with 0.5% bovine serum albumin and 2 mmol/EDTA) and stained with streptavidin-Qdot 655 (Invitrogen, Carlsbad, California, USA) for 30 minutes at 4°C, in the dark. Cells were washed again and run on a FACS CANTO 2.6 cytometer (BD Bioscences, San Jose, California, USA).

#### Intracellular staining

For the measurement of intracellular cytokine production, a separate aliquot of PBMCs was incubated with R10 (unstimulated), PMA and ionomycin (n = 40) or α-GalCer (n = 20), as described above. After 1 h at 37°C in 5% CO_2_, Brefeldin A (BRFA) (10 µg/mL; Sigma-Aldrich, Milan, Italy,) was added. After incubation for 14 h, the cells were washed and surface stained as described above, with the exception of anti-CD161. Cells were washed again and incubated with 1 mL of FACS Lysing Solutions (BD Bioscences, San Jose, California, USA) for 45 minutes at room temperature, in the dark and washed prior to intracellular staining with anti-TNF-α-FITC (BD Bioscences, San Jose, California, USA)/IFN-γ-FITC (Beckman Coulter, Fullerton, California, USA). After 30 minutes of incubation at 4°C in the dark, samples were washed, run on a FACS CANTO 2.6 cytometer and analyzed with FACS Diva 6.1.3 software.

### Statistical analysis

Data were analyzed with GraphPad 5 PRISM software. Fisher’s exact test, Chi-squared test, Mann-Whitney *U*-test, Kruskall-Wallis followed by Dunn’s post hoc analysis and Wilcoxon tests were used for statistics. Differences were considered statistically significant at p<0.05.

## Results

### Patient characteristics

Forty HIV-positive patients with available BMD and carotid IMT measurements were consecutively recruited at the Clinic of Infectious and Tropical Diseases, San Paolo Hospital, University of Milan. Subjects were divided into the following 4 groups (Table 1): 10 Double Positive (DP) patients; 10 Bone Disease (BD); 10 Cardiovascular Disease (CD); 10 Double Negative (DN) patients (Table 1). 3 patients in the BD group were diagnosed with osteoporosis and 7 subjects in the CD group presented a carotid plaque upon ultrasonography.

**Table 1:** Patient characteristics.

Characteristic	DP (n = 10)	BD (n = 10)	CD (n = 10)	DN (n = 10)
**Age, years (IQR)**	50 (44–57)	44 (38–62)	45 (43–54)	42 (38–50)
**Sex, F (%)**	3 (30)	3(30)	3 (30)	5 (50)
**Duration of HIV infection, mths (IQR)**	82 (67–323)	96 (44–183)	198 (141–263)	189 (71–225)
**HIV epidemiology, n (%)**				
MSM	2 (20)	2 (20)	3 (30)	4 (40)
Heterosex	6 (60)	3 (30)	3 (30)	6 (60)
IVD use	2 (20)	3 (30)	4 (40)	0 (0)
**HCV-Ab, n (%)**	1 (10)	3 (30)	3 (30)	0 (0)
**Nadir CD4, cells/mmc (IQR)**	89 (60–222)	210 (109–362)	232 (80–280)	321 (54–397)
**White blood cells (1000/mmc)**	5.6 (4.7–7.9)	5.6 (5.1–6.5)^c^	6.0 (5.4–8.1)^e^	4.7 (4.5–5.6)
Neutrophils (%)	56 (48.0–64.0)	56 (50.0–59.0)	56 (49.0–68.0)	52 (45.0–64.0)
Lymphocytes (%)	32 (26.0–39.0)	34 (30.0–40.0)	37 (24.0–41.0)	34 (26.0–40.0)
Monocytes (%)	7.8 (7.2–9.1)	7.2 (6.3–8.0)	8.2 (7.7–8.8)	9 (6.8–11.4)
Eosinophils (%)	2.7 (1.6–4.1)	1.8 (1.3–2.6)	1.9 (1.4–2.6)	1.6 (1.0–3.6)
Basophils (%)	0.6 (0.4–0.6)	0.7 (0.5–0.7)	0.5 (0.4–0.6)	0.5 (0.3–0.6)
Lymphocytes (n)	1928 (1375–2049)	1862 (1332–2179)	1919 (1675–2551)	1733 (1317–2239)
**Zenith HIV RNA, log10 cp/mL (IQR)**	5.4 (4.6–5.9)	5.6 (4.9–5.9)	5.3 (5.2–5.7)	5.0 (4.5–5.4)
**AIDS diagnosis n (%)**	4 (40)	3 (30)	0 (0)	3 (30)
**Current CD4 counts, cells/mmc (IQR)**	515 (308–575)	583 (379–716)	570 (517–798)	532 (354–614)
**Current CD4 counts, %**	26 (23–30)	33 (26–39)	32 (24–39)	30 (22–40)
**Current HIV RNA, log10 cp/mL (IQR)**	1.59	1.59	1.59	1.59
**HAART duration, mths (IQR)**	72 (63–149)	88 (45–123)	164 (56–180)	76 (52–130)
**HAART regimen**				
PI (%)	2 (20)	3 (30)	3 (30)	6 (60)
NNRTI (%)	6 (60)	6 (60)	6 (60)	4 (40)
Other (%)	2 (20)	1 (10)	1 (10)	0 (0)
Tenofovir use (%)	7 (70)	8 (80)	8 (80)	9 (90)
**DXA (IQR)**				
T-score femoral neck	−1.55 (−2.00−−1.10)^a,f^	−1.80 (−2.15−−1.35)^c,d^	−0.25 (−0.90−1.35)	−0.55 (−0.95−0.80)
T-score lumbar spine	−1.75 (−2.15−−1.48)^a,f^	−2.10 (−2.73−−1.45)^c.d^	−0.25 (0.25–1.08)	0.00 (−0.25−0.43)
**Carotid IMT, mm (IQR)**				
Left	1.04 (0.93–1.11)^a,b^	0.82 (0.65−0.96)	1.05 (0.98–1.21)^e^	0.85 (0.80−0.93)
Right	1.05 (0.96–1.25)^a^	0.87 (0.65−0.97)^c^	1.11 (1.00–1.38)^e^	0.89 (0.83−0.91)

DP: Double Positive; BD: Bone Disease; CD: Cardiovascular Disease; DN: Double Negative. MSM: Males Who Have Sex With Males. IVD: Intravenous Drug. HCV: Hepatitis C Virus. HAART: Highly Active Antiretroviral Therapy; PI: Protease Inhibitor; NNRTI: Non-Nucleoside Retroscriptase Inhibitor. DXA: Dual-energy X-ray absorptiometry. IMT: Intima Media Thickness. Data presented as: median (interquartile range, IQR) for continuous variables; absolute number (percentage) for categorical variables. p<0.05: ^a^DP vs DN; ^b^DP vs BD; ^c^BD vs DN; ^d^BD vs CD; ^e^CD vs DN; ^f^CD vs DP.

Study subgroups were comparable in terms of demographic and HIV-related parameters (Table 1).

Differences in BMD and IMT values were registered among groups as per inclusion criteria (Table 1).

### HIV-infected DP patients display high levels of CD161-expressing iNKT cells

**Figure 1:**
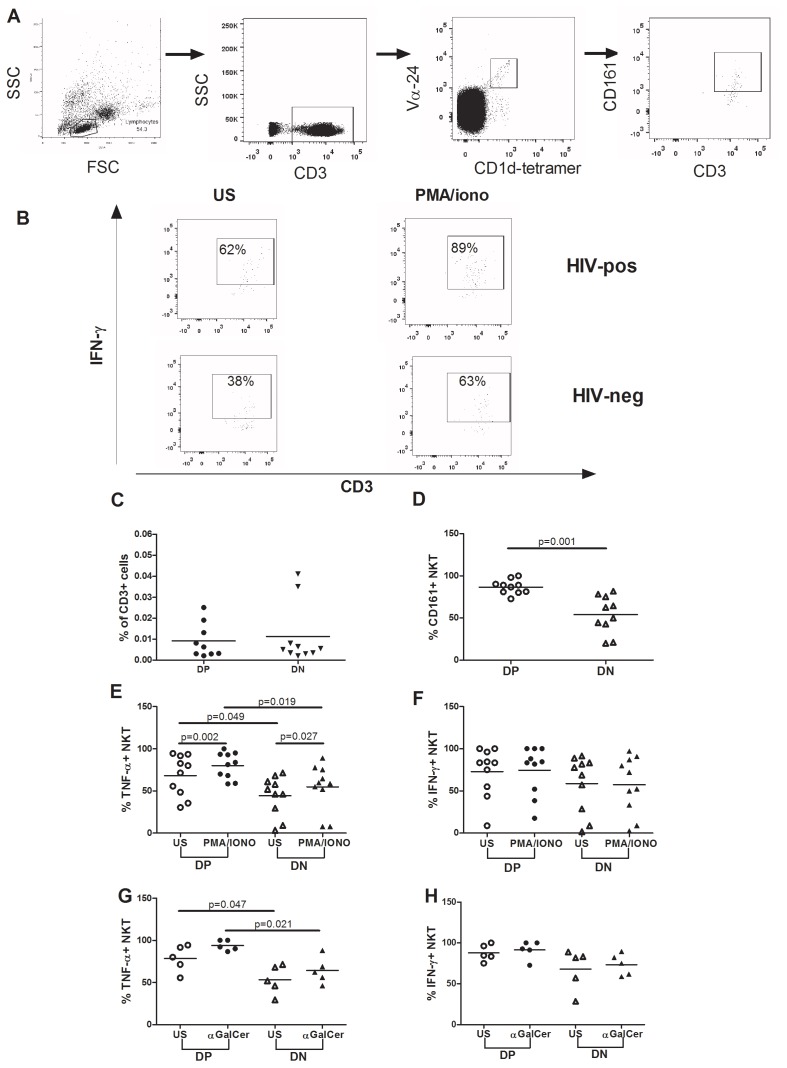
iNKT cell phenotype and function in HIV-positive “Double Positive” (DP) and “Double Negative” (DN) patients. Gating strategy of flow cytometry analysis for staining of iNKT cell frequencies, phenotype and intracellular cytokine production in a representative HIV-positive individual (A); an example of staining for intracellular cytokines is also shown of a representative HIV-negative subject (B). PBMCs were gated on lymphocytes, and iNKT cells were visualized as CD3+, Vα24+ and CD1d-tetramer+. An example of CD161 surface staining is shown in the far right plot. iNKT frequency were comparable in DP and DN groups (C). iNKT cell phenotype was analyzed through the *ex vivo* expression of CD161 in DP (n = 10) and DN (n = 10) patients (D). DP subjects exhibited significantly higher levels of CD161 on iNKT cell surface compared to DN patients (p = .001). iNKT cell function was measured through the production of TNF and IFN-γ *ex vivo* (US) and following stimulation with PMA/ionomycin (n = 10 per group) (E, F) and α-GalCer (n = 5 per group) (G, H). Although DP and DN patients significantly increased TNF production upon PMA/ionomycin stimulation (p = .002 and p = .027 respectively), DP subjects showed higher TNF release both prior to (p = .049) and following PMA/ionomycin (E). Study groups exhibited similar frequencies of IFN-γ-producing iNKT cells both *ex vivo* and after stimulation with PMA/ionomycin (F). DP patients were characterized by significantly higher TNF release both prior to (p = .047) and following stimulation with α-GalCer (p = .021) (G). Similar results were obtained in terms of IFN-γ production, with a trend to higher cytokine production in DP subjects following iNKT-specific stimulation (p = .059) (H). FSC, Forward Scatter: SSC, Side Scatter. Each symbol represents an individual.

We used a tetramer-based gating strategy (Fig. 1A) to measure iNKT cell frequencies in HIV-positive patients with bone and cardiovascular impairment (Double Positive, DP) and in HIV-infected subjects free from co-morbidities (Double Negative, DN).

The frequency of iNKT cells in peripheral blood was similar in the two patient groups (DP:.007% [IQR:.003–.021]; DN:.005% [IQR:.003–.015]; p = .88; Fig. 1C); interestingly, however, DP patients exhibited significantly higher CD161-expressing iNKT cells in comparison to DN patients (DP: 87.8% [IQR: 80.6–92] vs DN: 56.3% [IQR 37.4–76.1]; p = .001; Fig. 1D), suggesting an exhausted iNKT phenotype in HIV-infected subjects with bone and cardiovascular impairment.

### HIV-positive DP patients display high TNF production from iNKT cells both constitutively and following stimulation

Given that iNKT cells are able to produce a wide range of Th1 and Th2 cytokines, we determined the functional status of circulating iNKT by measuring TNF and IFN-γ directly *ex vivo* and after stimulation with PMA/ionomycin (Figure 1E, F) and the iNKT-specific stimulus α-GalCer (Fig. 1G, H).

Both DP and DN patients displayed a significant increase in TNF production following PMA/ionomycin (p = .002 and p = .027 respectively; Fig. 1E). Interestingly however, DP patients showed higher, spontaneous release of TNF both prior to stimulation (75.8% [IQR: 46.3–92.1] vs 49.1% [IQR: 24.4–62.9]; p = .049; Fig. 1E) and following PMA/ionomycin stimulation (DP: 82.6% [IQR: 65.9–93.9]; DN 59.4% [IQR: 41–76.1]; p = .019; Fig. 1E).

The functional capacity of iNKT in terms of INF-γ production did not differ between groups. Indeed, neither DP nor DN patients displayed a significant response to PMA/ionomycin (DP us: 83.3% [IQR: 52.2–87.1]; DP stim: DP: 84% [IQR: 48.6–100]; p = .58; DN us: 73.3% [IQR: 24–84.7]; DN stim: 62.2% [IQR: 27.2–88]; p = .38; Fig. 1F); moreover, INF-γ release from iNKT cells was comparable between subjects both prior to (p = .24) and following PMA/ionomycin (p = .19; Fig. 1F).

Given the differences in TNF production upon PMA/ionomycin, we decided to evaluate iNKT functional capacity in response to iNKT-specific stimulation with αGalCer in 5 patients per group.

In keeping with the above mentioned findings, significantly higher spontaneous TNF release was noted in DP patients (DP: 80% [IQR: 63.5–93.1]; DN: 52% [IQR: 37.8–69.8]; p = .047; Fig. 1G), who tended to significant TNF production after α-GalCer (p = .063). Accordingly, TNF release from α-GalCer-activated iNKT cells was greater in DP subjects (DP: 92.3% [IQR: 88.3–100]; DN: 62.3% [IQR: 51.1–78.5]; p = .021; Fig. 1G).

While no differences were observed in terms of IFN-γ production upon PMA/ionomycin, a non-significant trend to higher cytokine release was noted following α-GalCer stimulation in DP compared to DN subjects (92.9% [IQR: 82–100]; 75% [IQR: 60.3–89.5] respectively; p = .059; Fig. 1G).

Taken together, these findings suggest that iNKT cells from HIV-infected patients with concurrent bone and cardiovascular disease selectively produce high levels of TNF, both constitutively and upon stimulation.

### HIV-positive BD patients display normal iNKT cell frequencies and CD161 expression

Given that HIV-positive patients with bone and cardiovascular impairment present higher levels of CD161-expressing and TNF-producing iNKT cells, we aimed to investigate the phenotype and functional capacity of these cells in the two disorders separately.

We thus measured the frequency of total and CD161-expressing iNKT cells in patients with Bone Disease (BD) and compared them to patients free from co-morbidities (DN subjects).

**Figure 2:**
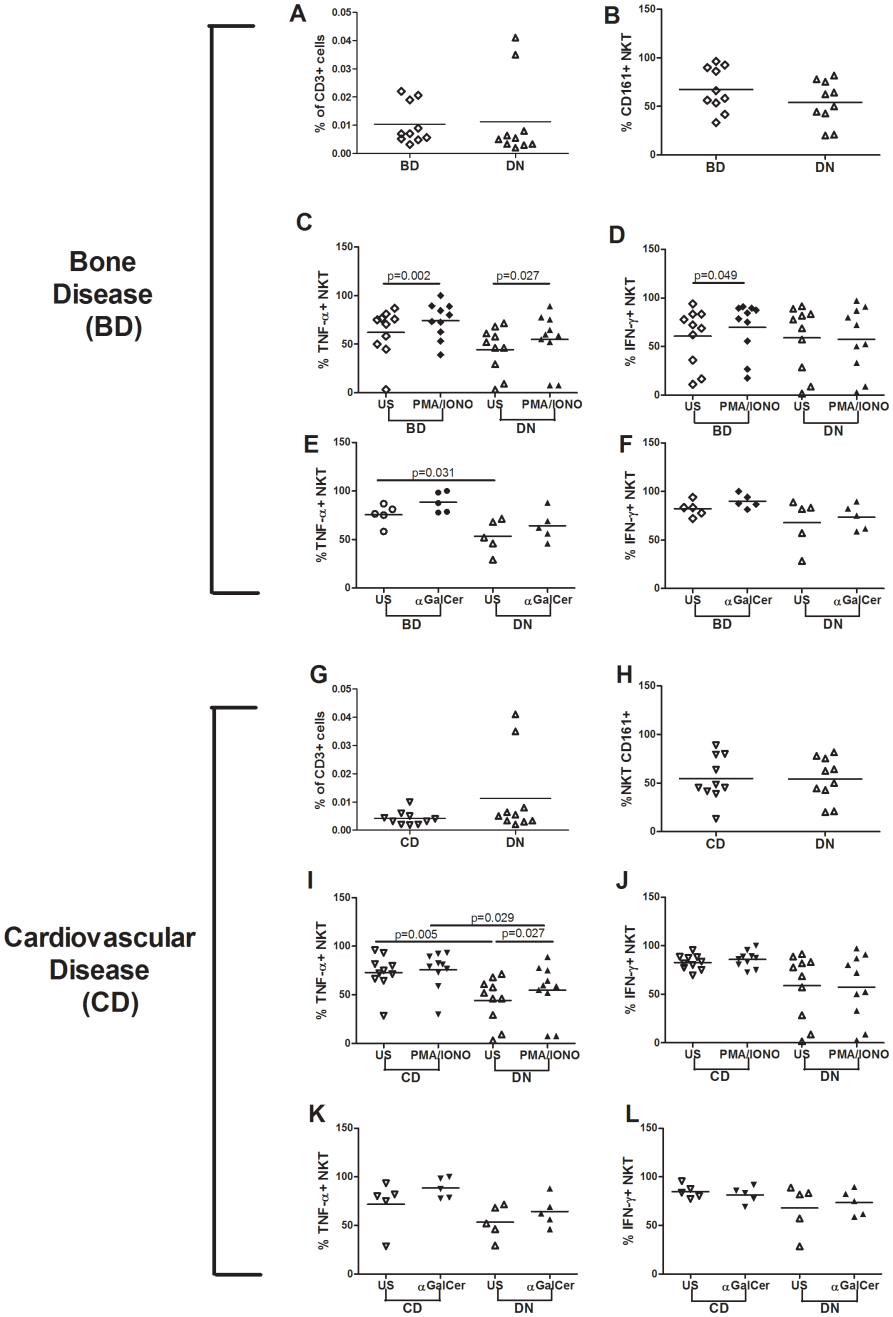
iNKT cell phenotype and function in HIV-positive “Bone Disease” (BD), “Cardiovascular Disease” (CD) and “Double Negative” (DN) patients. iNKT frequency was comparable among BD and DN groups (A). BD (n = 10) and DN (n = 10) showed similar CD161-expressing iNKT cell frequencies (B) and a significant increase in TNF production following PMA/ionomycin stimulation (p = .002 and p = .027 respectively). Despite a trend to higher spontaneous TNF release in BD patients (p = .075), comparable cytokine levels were recorded upon PMA/ionomycin (C). BD patients alone responded to PMA/ionomycin with significant IFN-γ production following stimulation (p = .0488) (D). Significantly higher TNF production was detected in BD subjects (p = .031) prior to α-GalCer stimulation. Upon α-GalCer stimulation, BD patients displayed a trend to significant increases in TNF release (p = .063), leading to higher cytokine levels in this population (p = .056) (E). No significant differences were noted in terms of IFN-γ production following α-GalCer, although BD patients tended to significant cytokine production (p = .063) (F). CD and DN showed comparable iNKT cell frequencies (G). CD (n = 10) and DN (n = 10) showed similar CD161-expressing iNKT cell frequencies (H). CD subjects showed higher TNF release both prior to (p = .005) and following stimulation with PMA/ionomycin (p = .029). Of note, DN patients alone responded to stimulation by significantly increasing TNF release from iNKT cells aspecific stimulation (p = .027) (I). In keeping with these results, the CD group displayed a trend to higher IFN-γ release after PMA/ionomycin stimulation (p = .052) (J). No statistical differences were noted between groups in terms of iNKT function following specific activation with α-GalCer (K, L). Horizontal lines indicate median values. Each symbol represents an individual.

Differently from what observed in patients with both bone and cardiovascular disease, the frequency of total (BD:.007% [IQR:.005–.019]; DN.005% [IQR:.003–.015], p = .34 Figure 2A), and CD161-expressing iNKT cells (BD: 62.3% [IQR: 50.7–90.7]; DN 56.3% [IQR 37.4–76.1]; p = .25; Fig. 2B) were comparable in the two groups.

### HIV-positive BD patients display high levels of TNF and IFN-γ from iNKT cells following stimulation

The functional capacity of iNKT cells in patients with bone disease was measured through the production of TNF and IFN-γ prior to and following stimulation with PMA/ionomycin (Fig. 2C, D) and α-GalCer (Fig. 2E, F).

Both BD and DN patients displayed a significant increase in TNF-producing cells following stimulation with PMA/ionomycin (BD us: 72.8% [IQR: 48.7–77.6]; BD stim: 76.7% [IQR: 60.2–89], p = .002: see above for DN values; Fig. 2C). A non-significant trend to higher spontaneous TNF release was observed in BD patients (p = .075) with no differences between study individuals upon PMA/ionomycin (p = .11) (Fig. 2C).

In reference to IFN-γ production, BD patients alone responded significantly to PMA/ionomycin (BD us: 70.5% [IQR: 31.1–83.3], BD stim: 81.6% [IQR: 48.3–89.4]; p = .049; Fig. 2D), with no differences among groups prior to and following stimulation (Fig. 2D).

Upon iNKT-specific α-GalCer activation, BD patients displayed a non-significant trend to an increase in TNF production (BD us: 76.5% [IQR: 66.7–84]; BD stim: 87.5% [IQR: 78.2–99.1]; (p = .063), (Fig. 2E), with a tendency to higher cytokine release compared to DN subjects (p = .056; see above for DN values) (Fig. 2E). Of note, the finding of higher spontaneous TNF release in the BD patient group prior to α-GalCer stimulation which was not detected prior to PMA/ionomycin may reflect the smaller sample size in the former experimental condition (n = 5 vs n = 10, respectively) (Fig. 2E).

Similarly, BD patients were characterized by a non-significant trend to higher IFN-γ production (DB us: 83.3% [IQR: 75–88.7]; BD: stim: 87.5% [IQR: 84.1–97.1]; p = .063; Fig. 2F) with no differences in cytokine release upon specific iNKT-activation (p = .095; Fig. 2F).

Taken together, the present findings suggest that HIV-positive subjects with bone impairment, display a tendency to a more activated functional profile of iNKT cells compared to DN individuals.

### HIV-positive CD patients display normal iNKT cell frequencies and CD161 expression

We next evaluated the frequency of total and CD161-expressing iNKT cells in HIV-infected patients with CD.

Similarly to what registered in patients with bone impairment alone, CD and DN subjects displayed similar iNKT frequencies (CD: 0.004% [IQR: 0.002–0.005]; see above for DN values p = .17: Figure 2G) and proportion of CD161-expressing cells (CD: 47.1% [IQR: 41–79.5]; see above for DN values p = 1.0) (Fig. 2H).

### HIV-positive CD patients display a highly activated and functionally exhausted iNKT-cell phenotype

iNKT cell function was measured *ex vivo* and following PMA/ionomycin (Fig. 2I, J) and α-GalCer stimulation (Fig. 2K, L).

Compared to DN subjects, patients with CD presented significantly higher TNF levels both prior to (CD us: 73.7% [IQR: 66.2–84.7]; DN see above; p = .005; Fig. 2I) and following mitogen stimulation (CD post PMA/ionomycin: 80.2% [IQR: 69.6–90.2]; DN see above p = .029; Fig. 2I). Of note however, healthy patients alone were able to induce significant TNF release upon PMA/ionomycin, suggesting a highly activated and exhausted iNKT phenotype in HIV-patients with cardiovascular disease.

Similarly, CD patients presented a non-significant trend to higher post-stimulation IFN-γ production from iNKT cells (86.9% [IQR: 79.3–90.8]; p = .052; Fig. 2J; see above for DN values) despite similar spontaneous cytokine release between groups (CD: 82.85% [IQR: 76.7–88.3]; see above for DN values p = .15; Fig. 2J).

The study of the functional capacity of iNKT cells following specific stimulation with α-GalCer revealed comparable TNF and IFN-γ production between subjects in all experimental conditions (Fig. 2K, 2L).

## Discussion

The objective of the present study was to assess whether HIV-positive individuals on virologically-suppressive HAART with bone and cardiovascular co-morbidities are characterized by specific iNKT cell phenotype and function.

The key finding of our analysis is the expansion of CD161-expressing and, TNF-secreting iNKT cells in subjects with coexisting bone and cardiovascular impairment; a less clear iNKT fingerprint was found in patients affected by single co-morbidities.

HIV-related T-lymphocyte activation has been postulated as a possible driving force of premature osteopenia/osteoporosis [13] and coronary heart disease [14]–[18]. HIV infection is also featured by impaired iNKT cell frequency [31], [33], [34], [36], [37] and function [32], [35]. In HIV-negative cohorts, iNKT cells have been involved in the pathogenesis of cardiovascular disease; recently a correlation between the loss of an anti-inflammatory subset of gut-residing CD4+ iNKT cells and systemic immune activation has been described in HIV infection [37], providing evidence for the contribution of this particular cell subset in the pathogenesis of HIV disease and non-communicable co-morbidities.

This is the first report to describe a peculiar iNKT phenotype in HIV-positive patients with concomitant early bone and cardiovascular disease, consisting of heightened CD161 expression. In our cohort, DP patients appear to be older and present a lower CD4 T-cell nadir, albeit not statistically significant when compared to the other subject groups; these findings are in line with literature data showing a higher prevalence of non-infectious co-morbidities in the aging population and in patients with a history of advanced HIV infection [10], thus reinforcing our findings on a peculiar iNKT phenotype in this setting. Interestingly, when analyzing subjects with either bone or cardiovascular impairment, we did not observe differences in terms of iNKT surface expression of CD161 between diseased and healthy individuals. Given that CD161 up-regulation reflects a more mature iNKT cell phenotype [56], [57], our finding suggests the preferential expansion of senescent circulating iNKT cells in HIV-infected patients with multiple co-morbidities and not in individuals with only one non-communicable disorder, although the smaller sample size may affect data analysis in the latter groups.

CD161 is also expressed on NK and CD8+ T cells [58]. Interestingly CD161+ NK cells have been reported to infiltrate the human atherosclerotic plaque [51]; to our knowledge, studies on CD161-expressing CD8+ T cells in bone and cardiovascular co-morbidities in course of physiological aging are currently lacking and it would be interesting to assess the role of this subset in these settings.

Heightened CD161 expression was also associated with increased iNKT TNF release in patients with bone and cardiovascular impairment. This is in contrast to previous reports describing an inverse correlation between CD161 and cytokine production from iNKT cells [32]; however participants in the present study were all on virologically-suppressive HAART and may have undergone iNKT restoration [38]–[40]. Indeed, our findings suggest that these cells are capable of producing high levels of TNF, which, in turn, may represent the major driver of iNKT-mediated inflammation and exacerbate bone/cardiovascular co-morbidities.

In order to evaluate the role of iNKT cells in the pathogenesis of bone and cardiovascular disease alone, we investigated their functional capacity in patients with either bone or cardiovascular impairment.

In keeping with the evidence of a pro-inflammatory milieu in the pathogenesis of non-infectious diseases in course of HIV [1], [10], [11], subjects with bone impairment showed a trend to higher spontaneous *ex vivo* TNF secretion and a significant IFN-γ increase release after stimulation, Given the limited number of patients enrolled and the weak statistical significance of such findings we cannot draw definitive conclusions on the role of iNKT cells in bone disease. iNKT cell expression of other members of the TNF superfamily such as RANKL, known to be involved in bone homeostasis, should be explored in such setting.

Finally, we concentrated on the role of iNKT cells in the pathogenesis of cardiovascular disease. Individuals with atherosclerosis displayed higher TNF secretion, both constitutively and after stimulation. However, upon stimulation, iNKT cells from patients with vascular damage were not able to further release TNF, possibly suggesting exhausted functional capacity.

In line with the role of IFN-γ–producing iNKT cells in the pathogenesis of atherosclerosis and plaque stability [45], [51], we also found a trend to higher IFN-γ secreting iNKT in patients with endothelial damage; this finding did not reach statistical significance, possibly due to limited sample size.

In the attempt to control for differences in antigen presenting cells in the PBMC mixtures, white blood cell count and formula were analyzed in our study population. The finding of similar percentages of circulating monocytes in all study patients suggests that iNKT phenotype and function may not be affected by diverse monocyte frequencies, albeit not ruling out possible differences other antigen presenting cells.

Several limitations exist in this study. First, the lack of HIV-negative controls; second, individuals with co-morbidities represent a heterogeneous population ranging from patients with preclinical damage (i.e. osteopenia/increased IMT) to subjects with overt disease (i.e. osteoporosis/carotid plaque). In this respect however, our findings were confirmed even when comparing patients with advanced disease (osteoporosis, n = 3; carotid plaque, n = 7) to individuals free from both co-morbidities. Further, our finding of similar iNKT function following mitogen and CD1d-antigen (α-GalCer) stimulation is in contrast to previous literature reports showing enhanced iNKT response to the former stimulus [32], and possibly reflects the different sample size used in the 2 experimental conditions. Finally the present work cannot establish a cause-effect relationship between activated iNKT and co-morbidities in the absence of a temporal relationship; moreover we cannot exclude that T-cell immune activation is driving the changes within the iNKT subset, as recently suggested by Fernandez et al. [59]. Future studies should also assess whether CD4- and CD8-expressing iNKT cells play a different role in the pathogenesis of non-infectious co-morbidities.

Despite these limitations, we show an increase in CD161-expressing and TNF secreting iNKT cells in HIV-positive individuals with bone and cardiovascular impairment, setting the basis for future studies specifically designed to investigate the role of iNKT cells in the pathogenesis of non-communicable co-morbidities in course of HIV infection.
